# Expression of Genes in the *16p11.2* Locus during Development of the Human Fetal Cerebral Cortex

**DOI:** 10.1093/cercor/bhab067

**Published:** 2021-04-07

**Authors:** Sarah Morson, Yifei Yang, David J Price, Thomas Pratt

**Affiliations:** Simons Initiative for the Developing Brain, Hugh Robson Building, Edinburgh Medical School Biomedical Sciences, The University of Edinburgh, Edinburgh EH8 9XD, UK; Centre for Discovery Brain Sciences, Hugh Robson Building, Edinburgh Medical School Biomedical Sciences, The University of Edinburgh, Edinburgh EH8 9XD, UK; Simons Initiative for the Developing Brain, Hugh Robson Building, Edinburgh Medical School Biomedical Sciences, The University of Edinburgh, Edinburgh EH8 9XD, UK; Centre for Discovery Brain Sciences, Hugh Robson Building, Edinburgh Medical School Biomedical Sciences, The University of Edinburgh, Edinburgh EH8 9XD, UK; Simons Initiative for the Developing Brain, Hugh Robson Building, Edinburgh Medical School Biomedical Sciences, The University of Edinburgh, Edinburgh EH8 9XD, UK; Centre for Discovery Brain Sciences, Hugh Robson Building, Edinburgh Medical School Biomedical Sciences, The University of Edinburgh, Edinburgh EH8 9XD, UK; Simons Initiative for the Developing Brain, Hugh Robson Building, Edinburgh Medical School Biomedical Sciences, The University of Edinburgh, Edinburgh EH8 9XD, UK; Centre for Discovery Brain Sciences, Hugh Robson Building, Edinburgh Medical School Biomedical Sciences, The University of Edinburgh, Edinburgh EH8 9XD, UK

**Keywords:** ALDOA, autism, cerebral cortex, CNV, KIF22

## Abstract

The 593 kbp *16p11.2* copy number variation (CNV) affects the gene dosage of 29 protein coding genes, with heterozygous *16p11.2* microduplication or microdeletion implicated in about 1% of autism spectrum disorder (ASD) cases. The *16p11.2* CNV is frequently associated with macrocephaly or microcephaly indicating early defects of neurogenesis may contribute to subsequent ASD symptoms, but it is unknown which *16p11.2* transcripts are expressed in progenitors and whose levels are likely, therefore, to influence neurogenesis. Analysis of human fetal gene expression data revealed that *KIF22*, *ALDOA*, *HIRIP3*, *PAGR1*, and *MAZ* transcripts are expressed in neural progenitors with *ALDOA* and *KIF22* significantly enriched compared to post-mitotic cells. To investigate the possible roles of *ALDOA* and *KIF22* proteins in human cerebral cortex development we used immunohistochemical staining to describe their expression in late first and early second trimester human cerebral cortex. KIF22 protein is restricted to proliferating cells with its levels increasing during the cell cycle and peaking at mitosis. ALDOA protein is expressed in all cell types and does not vary with cell-cycle phase. Our expression analysis suggests the hypothesis that altered neurogenesis in the cerebral cortex contributes to ASD in *16p11.2* CNV patients.

## Introduction

Large, recurrent Copy Number Variations (CNVs) are implicated in many neuropsychiatric disorders including autism spectrum disorders (ASD), epilepsy, intellectual disability (ID), and schizophrenia (McCarthy et al. 2009; Girirajan and Eichler 2010; Levy et al. 2011; Sanders et al. 2011; Malhotra and Sebat 2012). The *16p11.2* CNV (OMIM 611913) encompasses a 593 kb DNA sequence in the p11.2 region of human chromosome 16 (BP4-BP5). This region harbors 29 protein coding genes and is strongly linked to neurodevelopmental disorders (NDDs) including ASD (Kumar et al. 2008; Bijlsma et al. 2009; Rosenfeld et al. 2010; Shinawi et al. 2010; Zufferey et al. 2012). This *16p11.2* region is flanked by two homologous 147kbp sequences that arose after the evolutionary divergence of humans from other primates, generating a hot-spot for mis-aligned recombination that explains the high frequency of the *16p11.2* CNV in the human population and also the high frequency of de novo *16p11.2* CNV (Nuttle et al. 2016). In humans, the *16p11.2* microdeletion is associated with transient infant brain overgrowth (macrocephaly) and focal thickening of the cerebral cortex, while the *16p11.2* microduplication is associated with reduced brain size (microcephaly) (Qureshi et al. 2014; Blackmon et al. 2018). The early manifestation of anatomical phenotype in newborns, along with the onset of ASD symptoms in infancy, suggests crucial roles for *16p11.2* genes during neural development. *16p11.2* is the most prevalent CNV associated with ASD, approximately 1% incidence, making this CNV particularly intriguing and providing motivation for investigating the role played by *16p11.2* genes in brain development and function (Weiss et al. 2008). Available lines of evidence from *16p11.2* rodent models, *16p11.2* patient derived lymphoblastoid cell lines, and human induced pluripotent stem cells genetically engineered to harbor the *16p11.2* CNV indicate that all *16p11.2* messenger RNAs’ (mRNAs’) levels reflect the altered gene dosage of *16p11.2* genes (50% in microdeletion and 150% in microduplication heterozygotes) (Horev et al. 2011; Blumenthal et al. 2014; Pucilowska et al. 2015; Tai et al. 2016). This indicates that multiple *16p11.2* transcript levels are affected by the *16p11.2* CNV and that the pathology of the *16p11.2* CNV could stem from altered dosage of one or more them.

Although none of the individual *16p11.2* genes have been identified as sole causative genes for the *16p11.2* phenotype, *MAPK3*, *QPRT, KCTD13*, *ALDO*A, *TAOK2*, and *KIF22* have each been individually associated with a variety of neural phenotypes in non-human models. These include cell proliferation, neuronal morphology, axonal projection and spine morphogenesis, altered head size, and behavioral phenotypes (Blaker-Lee et al. 2012; de Anda et al. 2012; Golzio et al. 2012; Pucilowska et al. 2015, 2018) (Escamilla et al. 2017) (Richter et al. 2019) (Yadav et al. 2017) (Ultanir et al. 2014).

The cellular mechanisms by which the *16p11.2* CNV causes the patient phenotype are poorly understood. One plausible hypothesis is that disrupted neurogenesis causes changes in neuronal output, which produce a brain with abnormal cell number or composition and that this contributes to the *16p11.2* pathology. Consistent with this hypothesis, the *16p11.2* deletion mouse model exhibits proliferation defects in cortical progenitors during prenatal brain development and subsequently develops ASD-like symptoms (Horev et al. 2011; Pucilowska et al. 2015). However, it is unknown which of the proteins produced by *16p11.2* CNV genes are expressed by progenitor cells in the developing human cerebral cortex and are therefore candidates for regulating neurogenesis.

Excitatory (glutamatergic) neurons in the human cerebral cortex are derived from progenitors that reside in the ventricular and subventricular zones of the dorsal telencephalon while inhibitory (GABAergic) interneurons are derived from progenitors that reside in the ventral telencephalon and migrate into the cerebral cortex (Clowry et al. 2010) (Bystron et al. 2008) (Ma et al. 2013) Here we focused on the potential for the *16p11.2* CNV to affect neurogenesis of excitatory neurons in the developing human cerebral cortex by identifying *16p11.2* genes that are highly expressed in cerebral cortex progenitors in the ventricular zones and down-regulated as cells become post-mitotic. We analyzed previously published human fetal cortex single cell RNA sequencing (scRNA-seq) data (Pollen et al. 2015) (Zhong et al. 2018) to identify candidate genes and characterize their expression in sections of developing human fetal cerebral cortex from the late first and early second trimester.

## Material and Methods

### Human Tissue

Human embryos ranging in age from 12 to 16 post-conceptual weeks (PCW) were obtained from the Medical Research Council (MRC)/Wellcome-Trust funded Human Developmental Biology Resource at Newcastle University (HDBR, http://www.hdbr.org/) with appropriate maternal written consent and approval from the Newcastle and North Tyneside NHS Health Authority Joint Ethics Committee. HDBR is regulated by the UK Human Tissue Authority (HTA; www.hta.gov.uk) and operates in accordance with the relevant HTA Codes of Practice. For cryosections 12 PCW week brains were fixed in 4%PFA/PBS for 1 week then cryoprotected with 30% sucrose/PBS and then embedded in 50:50 30%sucrose: OCT, flash frozen and sectioned at 12 μm using a Leica Cryostat. Stages used for this study: 12 PCW (two brains), 14 PCW (one brain) and 16 PCW (one brain).

### scRNA-seq Analysis

The publicly available scRNA-seq data sets (Pollen et al. 2015; Zhong et al. 2018) were used to identify candidate genes. Prior to dataset publication the reads were aligned, we normalized the RPKMs as log (x + 1). Analysis was performed using R studio. To determine genes with significant changes a Wilcox test by *FindAllMarkers* in Seurat package was used. Monocle2 R package was used to order cells in pseudotime. To identify cell-cycle phase specific transcripts we used function CellCycleScoring from Seurat R package.

### Immunohistochemistry

Immunohistochemistry was carried out on paraffin sections obtained from HDBR. Antigen retrieval consisting of boiling sections in 10 mM sodium citrate pH 6 for 10 min was used for all stains. Primary antibodies were diluted in 20% blocking serum in pH 7.6 Tris buffered saline (TBS) and sections incubated overnight at 4°C. Primary antibodies used: KID 1/5000 DAB, 1/2000 fluorescent (Invitrogen PA5–29490), KI67 1/800 (Novus Biologicals NBP2-22112), ALDOA 1/100 (Sigma HPA004177).

For colourmetric stains, sections were incubated 1 h at room temperature with biotinylated secondary antibody (1/200) followed by incubation for 1 h with ABC (Vector Labs) and developed with diaminobenzidine solution (Vector Labs), washed, counterstained with nuclear fast red, dehydrated and then mounted using DPX.

For immunofluorescence sections were incubated with secondary antibodies 1/200 1 h room temperature, counterstained with 4′,6-diamidino-2-phenylindole dihydrochloride (DAPI;ThermoFisher) and mounted with Vectashield H1400 Hardset Mounting Medium (Vector Labs). Extensive TBS washes were carried out between each step.

### In Situ Hybridisation

PCR primers used to clone in situ probes from human cDNA into pGEMTeasy for preparation of DIG labeled RNA were as follows: *ALDOA*, CTG TCA CTG GGA TCA CCT T, & GTG ATG GAC TTA GCA TTC AC*; KIF22*,: CGA GAG CGG ATG GTG CTA AT &: GAG ACC CAG GAT GTT TGC CT; *PAGR1*, ATG ATG AGC CAG TGA CAC CA & TCT GCC TCT CCC TTC AAG TG; *HIRIP3*, TGG TGC CCA TCG AAA CTA CA & TGG CCC AAA ATA CAG GAG GT; & *MAZ*, CAC GAG GAG AAA GTG CCA TG & GAG AGA AGA GGA CCG TCG AG.

In situ hybridisation was performed on cryosections of 12 PCW brain as described previously (Radonjić et al. 2014). Briefly, 12 μm cryosections were dried at 37 °C for 3 h then incubated overnight at 70 °C in hybridization mix containing ×1 salts (200 mM NaCl, 10 mM Tris HCl (pH 7.5), 1 mM Tris Base, 5 mM NaH_2_PO_4_2H_2_O, 5 mM Na_2_HPO_4_, 0.5 M EDTA: Sigma-Aldrich), 50% deionized formamide, 10% dextran sulfate, 1 mg/mL rRNA, ×1 Denhardt’s, and DIG-labeled RNA probe. Next day sections were washed three times at 70°C in wash buffer comprising ×1 SSC, 50% formamide, 0.1% Tween-20 and then three times at RT in ×1 MABT (20 mM Maleic acid, 30 mM NaCl, 0.5% Tween-20 and pH adjusted to 7.5 with 10 mM NaOH). Sections were incubated 1 h RT in ×1 MABT blocking solution (20% sheep serum, 2% blocking reagent) and then incubated overnight with anti-DIG antibody 1:1500 in blocking solution at 4°C followed by color reaction overnight at RT.

### Microscopy and Imaging

DAB and in situ hybridisation images were taken using a Leica DMNB microscope with an attached Leica DFC480 Camera. Fluorescence images were obtained with a Leica DM5500B epifluorescence microscope with a DFC360FX camera. Confocal images were obtained using Nikon A1R FILM microscope and analyzed in ImageJ.

### Image Analysis and Quantification

For DAB stains and in situ hybridisation the images were stitched in ImageJ using the stitching plugin (Preibisch et al. 2009).

For KIF22 analysis of DAB stains rectangular counting boxes (34x88μm) were overlaid across the section. Using ImageJ cell counting plugin cells in each box were counted and denoted KIF22+ (brown) or KIF22- (red). The distinction between the regions (VZ, SVZ, and IZ/CP) was determined anatomically by cell density. The count for each box was averaged with other boxes in the region to provide the final value.

For analysis of KIF22/KI67 double staining counting boxes (×20145 μm) were overlaid over the VZ and SVZ (determined based on cell density). For determining intensity cells were randomly selected on the DAPI channel, the nucleus outlined and intensity of KIF22 and KI67 recorded. 20 cells were selected per box and the counts from individual boxes combined to give final values.

For subcellular ALDOA analysis counting boxes (20 × 145 μm) were overlaid over the VZ and SVZ. Cells were randomly selected on the DAPI channel, far enough apart to ensure their cytoplasm would not overlap, the Z plane through the center of the cell was used and the nucleus outlined. The KI67 and ALDOA intensity was measured constituting the nuclear value. To obtain ALDOA cytoplasmic intensity the nuclear outline was duplicated and extended 4 pixels allowing a reading of just the cytoplasmic area to be obtained (see Fig. 6*e*). This was performed for 10 cells in each box and the counts from individual boxes combined to give final values.

### Data Analysis and Statistics

Where error bars are shown they are expressed as mean ± SEM. Boxplots show median and upper and lower quartiles. Statistical comparison between two groups was performed with a *t* test. Statistical comparison between more than two groups was performed with ANOVA followed by *post hoc* test. *P <* 0.05 was considered statistically significant. Analysis was performed using GraphPad Prism.

## Results

### Analysis of scRNA-seq Data Identifies *KIF22* and *ALDOA* as Progenitor-Enriched *16p11.2* Transcripts in the Developing Human Fetal Cerebral Cortex

**Figure 1:**
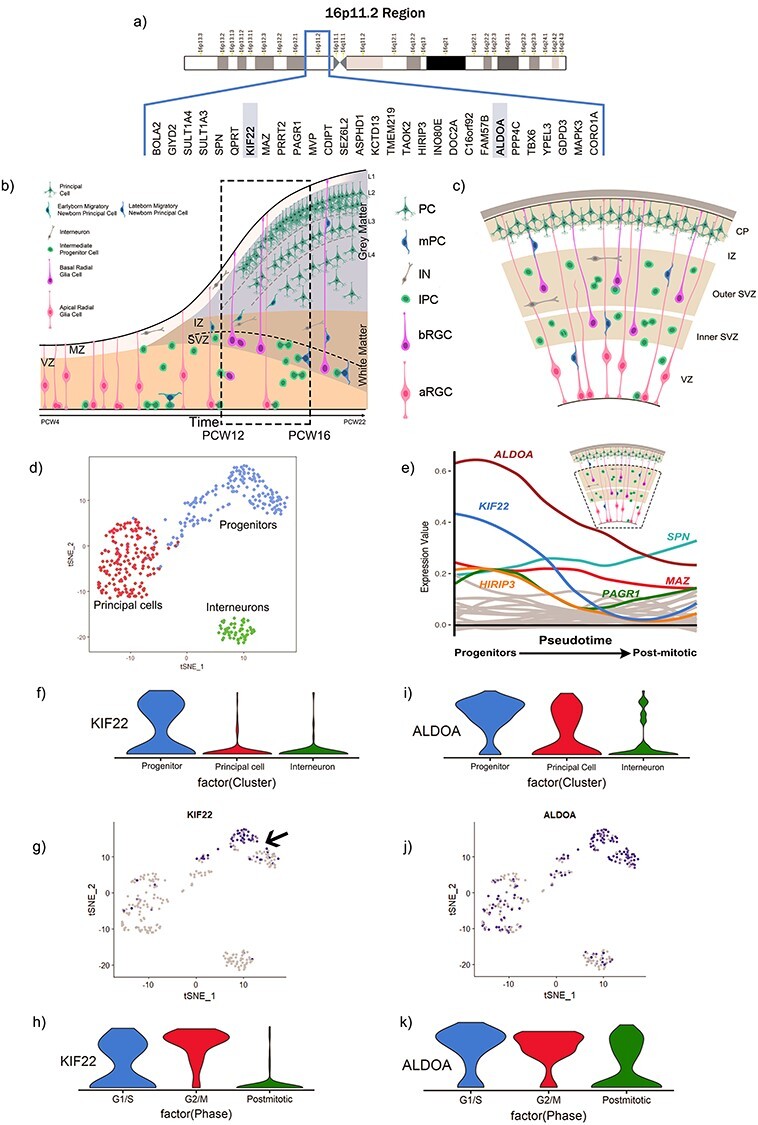
Bioinformatics analysis of scRNA-seq from the VZ and SVZ of the 16-18GW human fetal cortex. (*a*) 16p11.2 region and genes. (*b*) schematic adapted from Budday et al. 2015 shows the process of human fetal cortex development over time. Dotted box indicates approximately the time-period of interest for our study; 12-16PCW. (*c*) schematic of human cortical structure during development. (*d*) tSNE clustering of cell types. (*e*) changing mRNA expression levels of *16p11.2* genes as cells move from progenitors to neurons with *KIF22* and *ALDOA* transcripts identified as changing significantly. Schematic of human cortex with dotted box indicates the compartments of the brain that were used to generate this dataset—the germinative VZ and SVZ. (*f*) Violin plots showing distribution of *KIF22* in different cell types. (*g*) *KIF22* gradient plot (arrow indicates progenitors with a subset expressing high levels of KIF22 mRNA). (*h*) Violin plots showing distribution of *KIF22* at different cell cycle stages. (*i*) Violin plots showing distribution of *ALDOA* mRNA levels in different cell types. (*j*) *ALDOA* gradient plot. *k*) Violin plots showing distribution of *ALDOA* mRNA at different cell cycle stages.

The *16p11.2* CNV involves microduplication or microdeletion of a 593 kb locus on human chromosome 16 containing 29 protein coding genes (Fig. 1*a*). The aim of the current study is to identify *16p11.2* genes that are potential candidates for being involved in neurogenesis in the developing human cerebral cortex (Fig. 1*b,c*) and whose altered dosage in *16p11.2* microdeletion or microduplication patients may disrupt neurogenesis and contribute to the CNV phenotype. We reasoned that *16p11.2* genes important for neurogenesis would be highly expressed in proliferating progenitor cells and downregulated as cells became postmitotic.

We took advantage of a published single cell RNA-sequencing (scRNA-seq) data-set acquired from 393 cells of the ventricular zone (VZ) and subventricular zone (SVZ) of gestational week (GW) 16–18 human fetal cerebral cortex (equivalent to post conception week (PCW) 14–16) to perform an unbiased screen to identify *16p11.2* transcripts that matched this expression profile (Pollen et al. 2015). Dimensional reduction of the scRNA-seq data separated the cells into three clusters based on transcriptome similarity (Fig. 1*d*—each dot on the tSNE plot represents an individual cell) that were subsequently identified as the three cardinal cell classes of progenitors (blue), post-mitotic neurons/principal cells (red) and interneurons (green), by expression of cell-type specific transcripts. We next used the monocle2 R package to order the cells in pseudotime using the normalized expression levels of selected differentially expressed genes (DEGs) as input to order the cells (Trapnell et al. 2014; Qiu et al. 2017) (Fig. 1*e*) moving from the progenitor state (left) to post mitotic state (right) along the X-axis. We plotted the average expression of each *16p11.2* transcript at each pseudotime-point on the Y-axis. We found that two genes, *KIF22* (blue line) and *ALDOA* (brown line), were notable for having high expression in progenitors that declined as cells became post-mitotic. A Wilcox test identified *ALDOA* and *KIF22* as the only *16p11.2* transcripts that were significantly higher in progenitor than neuronal populations (*P* <  < 0.05). Although not significantly enriched in progenitors *HIRIP3* (orange line), *MAZ* (red line), and *PAGR1* (green line) were expressed in progenitors at higher levels than the remaining *16p11.2* transcripts (shown as gray lines), many of which were barely expressed at all.

Violin plots of the numbers of cells expressing different levels of *KIF22* mRNA in the different cardinal cell classes show that *KIF22* is expressed predominantly in progenitors (Fig. 1*f*) and mapping the expression level of *KIF22* onto the tSNE plot (Fig. 1*g*) revealed that *KIF22* expression is highest in a subset of the progenitor cluster (arrow in Fig. 1*g*) with a substantial proportion of progenitor cells expressing relatively low levels of *KIF22.* Very few post-mitotic neurons, both interneurons and principal cells, express appreciable levels of *KIF22* (Fig. 1*f,g*). The expression of *KIF22* in a subset of progenitors prompted us to ask whether its expression was related to the cell-cycle phase. We used the expression of cell-cycle phase specific transcripts using function CellCycleScoring from Seurat R package to divide the cells into three classes (Macosko et al. 2015; Tirosh et al. 2016), G1/S, G2/M, and post-mitotic neurons, and compared *KIF22* transcript levels between these three groups using a violin plot (Fig. 1h). We found that the majority of cells in G2/M phase expressed higher levels of *KIF22* (red plot), cells in G1/S expressed lower levels (blue plot) while the vast majority of post-mitotic cells expressed low levels of *KIF22* (green plot). Similar analysis for *ALDOA* show that while a greater proportion of cells expressing the highest levels of *ALDOA* are progenitors (blue plot) there are also a substantial number of principal cells (red plot) expressing similarly high levels of *ALDOA* transcripts although very few interneurons (green plot). Mapping *ALDOA* expression level onto the tSNE plot (Fig. 1*j*) shows cells expressing high levels of *ALDOA* are evenly distributed throughout the progenitor cluster with appreciable numbers of principal cells expressing high levels of *ALDOA* and a much lower proportion of interneurons. In contrast to *KIF22*, there is no clear difference in the partitioning of *ALDOA* expression level between different phases of the cell-cycle (Fig. 1*k*). We performed the same analysis on another developing human cortex scRNAseq data set that spanned a wider developmental interval (PCW10–28) and also included cells from all layers of the cerebral cortex (Zhong et al. 2018). This analysis produced similar results. *KIF2*2 transcripts showing them to be enriched in progenitors (Supplementary Figure 1*b, e*) and in G2/M phase of the cell cycle (Supplementary Fig. 1*g*). Although *ALDOA* transcript levels were highest in progenitors they did not decline as much as *KIF22* (Supplementary Fig. 1*b,f*) in postmitotic cells and showed no clear difference between different phases of the cell-cycle (Supplementary Fig. 1*h*).

**Figure 2:**
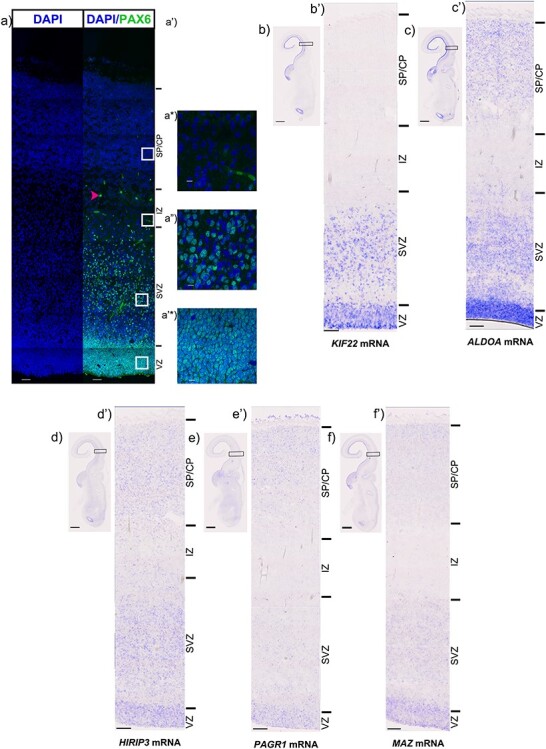
In situ hybridisation of candidate genes. (*a*) PAX6 protein (green) at 12pcw. a-a’^*^) show high magnification images of PAX6 protein expression in the (*a’*) SP/CP, a^*^) IZ, *a”)* SVZ and a’^*^) VZ. Low magnification scale bars = 50 μm, high magnification scale bars = 10 μm. b) Low magnification image of *KIF22* mRNA in the 12pcw human fetal cortex, *b’*) High magnification showing *KIF22* mRNA (blue) to be predominantly expressed in the germinative zones. *c*) Low magnification image of *ALDOA* mRNA in the 12pcw human fetal cortex, (*b’*) High magnification showing *ALDOA* mRNA (blue) to be predominantly expressed in the germinative zones but also some expression in the IZ and CP. (*d*) Low magnification image of *HIRIP3* mRNA in the 12pcw human fetal cortex, (*d’*) High magnification showing *HIRIP3* mRNA (blue) to be expressed throughout the telencephalic wall. *e*) Low magnification image of *PAGR1* mRNA in the 12pcw human fetal cortex, *e’)* High magnification showing *PAGR1* mRNA (blue) to be expressed throughout the telencephalic wall. (*f*) Low magnification image of *MAZ* mRNA in the 12pcw human fetal cortex, (*f’*) High magnification showing *MAZ* mRNA (blue) to be expressed throughout the telencephalic wall. For ISH, low magnification scale bars = 2 mm and high magnification scale bars = 100 μm.

We next used in situ hybridisation to visualize the expression of *KIF22*, *ALDOA*, *HIRIP3*, *PAGR1*, and *MAZ* transcripts in the different layers of the 12 PCW brain. We identified the VZ and SVZ based on cytoarchitecture, however, to validate our delineation we stained a section with PAX6, expressed by progenitor cells (Fig. 2*a*-a’^*^). This confirmed the absence of progenitor cells in the IZ and CP and allowed us a guidance for estimating cellular location in other sections.

Consistent with the scRNAseq data *KIF22* and *ALDOA* transcripts are the most clearly differentially expressed between zones containing progenitors (VZ and SVZ) and the more superficial layers that are mainly composed of postmitotic cells (IZ and SP/CP). *KIF22* mRNA expression is most prominent in the VZ and SVZ with a few expressing cells in the IZ and SP/CP (Fig. 2*b,b′*). Although *ALDOA* mRNA expression is most prominent in the proliferative VZ and SVZ there are substantial numbers of *ALDOA* expressing cells in the SP/CP (Fig. 2*c,c′*). *HIRIP3* (Fig. 2*d,d′*), *PAGR1* (Fig. 2*e,e′*), and *MAZ* (Fig. 2*f,f′*) mRNAs are expressed in the VZ/SVZ and also in cells of the SP/CP. In addition to being expressed in the proliferative zones of the cerebral cortex *KIF22*, *ALDOA*, *HIRIP3*, *PAGR1*, and *MAZ* are also expressed in the ventricular zone of the ganglionic eminences where interneuron progenitors reside suggesting the hypothesis that interneuron development may be affected by alterations in their dosage in the *16p11.2* CNV.

To conclude, of all the 29 *16p11.2* transcripts, five, *KIF22*, *ALDOA*, *HIRIP3*, *PAGR1*, and *MAZ*, are expressed in the ventricular and subventricular zones at higher levels than in post-mitotic cells. Of these only two*, KIF22* and *ALDOA*, are significantly enriched in progenitors compared to post-mitotic cells making them candidates for having specific roles in neurogenesis in the developing human fetal cerebral cortex. Although both are enriched in progenitors, *KIF22* and *ALDOA* transcript expression shows notable differences: *KIF22* transcripts are more restricted to progenitors and their levels vary as the cell-cycle progresses. We next describe the expression of KIF22 and ALDOA protein over a range of developmental stages.

### KIF22 Protein is Expressed in Germinal Zones of 12, 14, and 16 PCW Cortex

**Figure 3:**
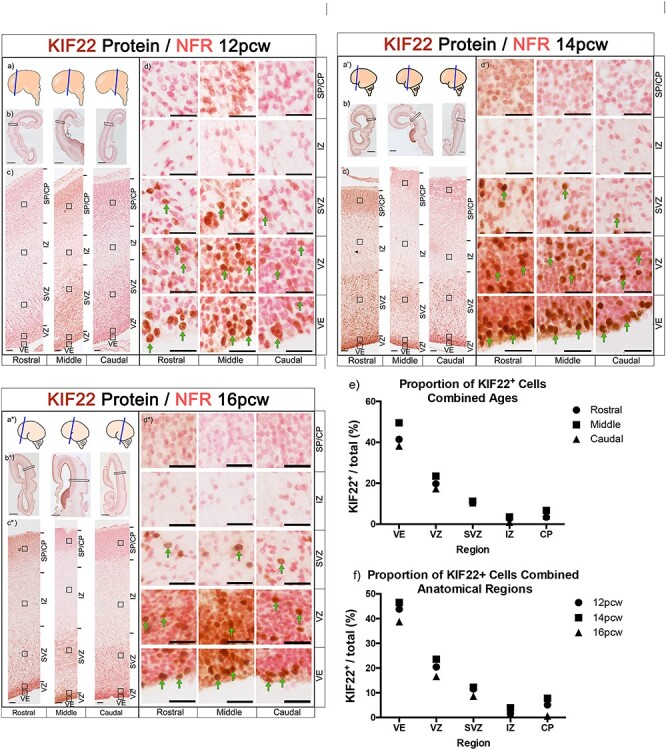
KIF22 protein expression levels in the cerebral cortex at 12, 14, and 16 PCW (a, a’, a^*^) schematic showing brain regions sectioned. (b, b’, b^*^) images of whole brain section, scale bars =2 mm. c, c’, c^*^) sections spanning the rostral-caudal axis showing KIF22 expression in the telencephalic wall, scale bars = 100 μm. (d, d’, d^*^) high magnification images of different cortical zones rostral-caudal. KIF22+ cells in brown and examples indicated by green arrows, KIF22^−^ cells in pink, scale bars =25 μm. (*e*) Quantification of KIF22 expressing cells with all three ages combined. (*f*) Quantification of KIF22 expressing cells with rostral, middle, caudal values combined.

Here we characterize KIF22 protein expression during human corticogenesis. Coronal cortex sections spaced along the rostral-caudal axis were immunostained for KIF22 protein and counterstained with Nuclear Fast Red (NFR) to show cytoarchitecture. KIF22^+^ (brown) and KIF22^−^ (red) cells were counted for each region in the telencephalic wall (VE, VZ, SVZ, IZ, and CP) (see methods for details of sampling) and lamination was identified by cell density (Bayer and Altman 2002, 2005). These data are shown for three developmental stages, 12 PCW (Fig. 3*a–d*), 14 PCW (Fig. 3*a′–d′*), and 16 PCW (Fig. 3a^*^–d^*^). At all stages and rostro-caudal positions KIF22 expressing cells appear most abundant in the VE followed by the VZ and SVZ with the IZ and CP presenting a very low to complete absence of KIF22 (Fig. 3*c,c′*,c^*^ with higher magnification of boxed regions from each zone shown in *d, d′*, and d^*^ respectively, green arrows indicate examples of individual KIF22^+^ cells).

We next pooled KIF22^+^ cell count data in two ways to compare between all ages (Fig. 3*e*) and anatomical regions (Fig. 3*f*) and found that the percentage of KIF22^+^ cells in the VE (40–50%) was consistently higher than other regions, followed by the VZ (20–30%) and SVZ (10%), with even fewer cells (<10%) in the IZ, and CP (Fig. 3*e,f*). This result describes KIF22 protein as predominantly restricted to a subset of cells in the germinal zones of the developing cortex at all stages studied.

### KIF22 Protein Expression is Restricted to Proliferating Cells

**Figure 4:**
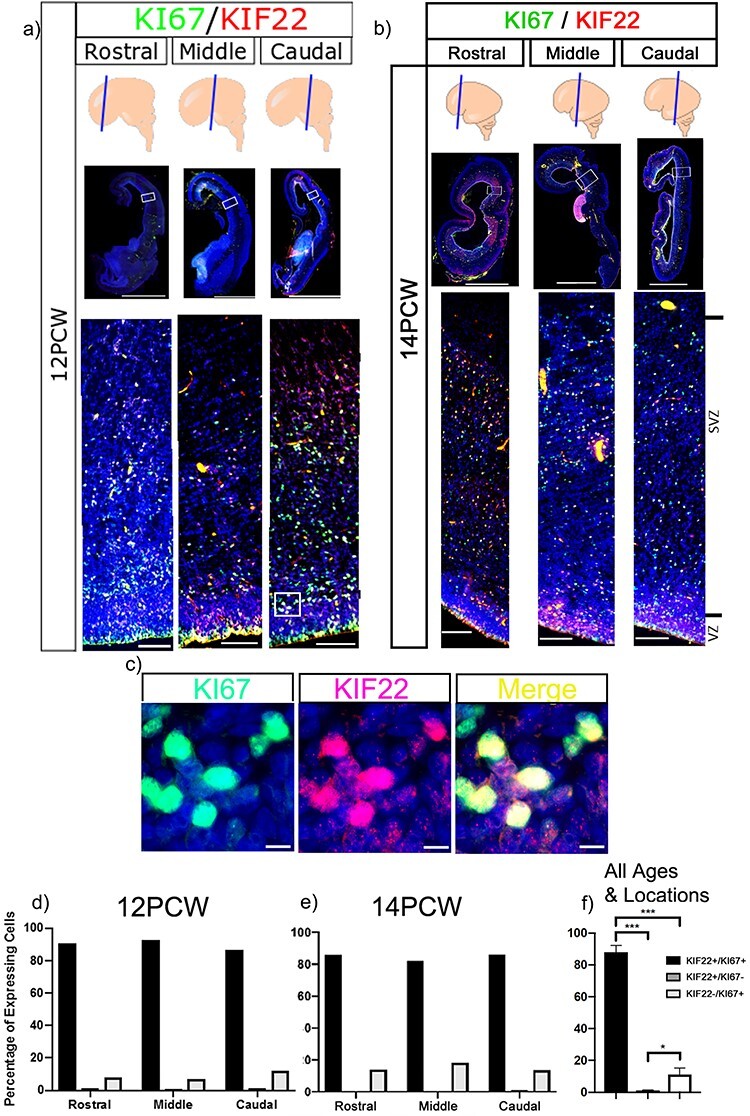
Immunofluorescence of KIF22 and KI67 proteins in the cortex. (*a*) KIF22 and KI67 at 12 PCW, low magnification scale bars = 4 mm, high magnification scale bars = 100 μm. (*b*) KIF22 and KI67 at 14 PCW, low magnification scale bars = 4 mm, high magnification scale bars = 100 μm. *c*) high magnification of KI67/KIF22 expressing cells. Scale bars = 10 μm. (d) Percentage of cells expressing KIF22, KI67, or both at 12 PCW. (*e*) Percentage of cells expressing KIF22, KI67, or both at 14 PCW. (*f*) Combined data of percentage of cells expressing KIF22, KI67, or both.

KIF22 protein expression is almost exclusively restricted to a subset of cells in the proliferative regions. From the scRNA-seq data, we expect these to be progenitor cells (Fig. 1). To identify these KIF22 positive cells we performed double immunofluorescence for KIF22 and KI67 (a protein expressed in all proliferating cells (Scholzen and Gerdes 2000; Miller et al. 2018)). KIF22^+^ cells were predominantly located in the VE, VZ, and SVZ (Fig. 3), therefore these regions were examined for analysis. Low magnification of KI67/KIF22 staining is shown in 12 PCW (Fig. 4*a*) and 14 PCW (Fig. 4*b*) with higher magnification showing individual cells in Fig. 4*c*. Cell counts for KIF22^+^/KI67^+^-labeled cells show that the majority (80–90%) of KI67^+^ cells also express KIF22 both at 12 (Fig. 4*d*) and 14 PCW (Fig. 4*e*) or across the rostral-caudal axis. Combining the data for anatomical locations and ages revealed significantly more KIF22^+^/KI67^+^ cells than KIF22^+^/KI67^−^ and KIF22^−^/KI67^+^ cells (Fig. 4*f*).

### KIF22 Levels Vary with Cell-Cycle Phase

**Figure 5:**
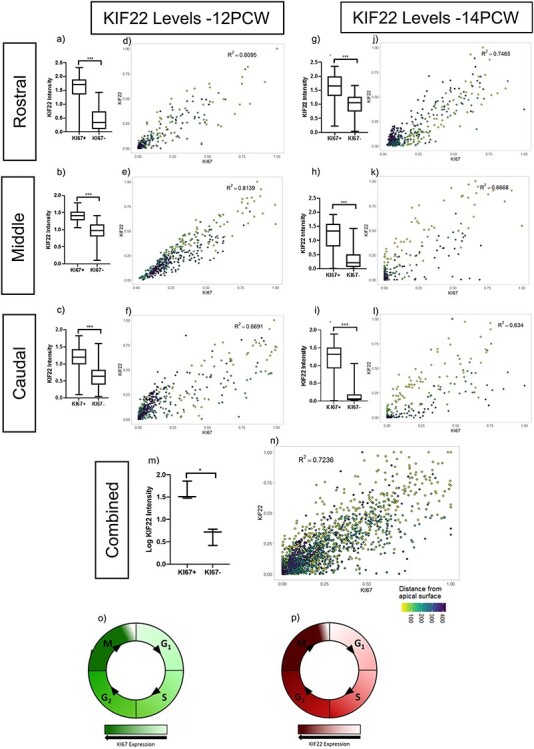
Quantification of KIF22 protein levels. (*a, b, c*) 12 PCW quantification of KIF22 fluorescence intensity in KI67+/KI67- cells (raw data transformation = +1(log), unpaired t-test with Welch’s correction,*P* = < 0.001). (*d, e, f*) intensity correlations of KIF22 and KI67 nuclear fluorescence intensity at 12 PCW. (*g, h, i*) 14 PCW quantification of KIF22 fluorescence intensity in KI67+/KI67- cells (raw data transformation = +1(log), unpaired *t*-test with Welch’s correction, *P* = < 0.001). *j, k, l*) intensity correlations of KIF22 and KI67 nuclear fluorescence intensity at 14 PCW. *m*) quantification of KIF22 fluorescence intensity in KI67+/KI67-cells 12 and 14 weeks combined (raw data transformation = (log), paired *t*-test, *P* = 0.0122). n) intensity correlations of KIF22 and KI67 nuclear fluorescence intensity for rostral-caudal points at 12 and 14 PCW with distance from apical surface indicated by dot color. *o*) diagram of KI67 protein levels throughout the cell cycle. *p*) model based on our results of KIF22 protein levels throughout the cell cycle.

From the scRNA-seq analysis, and the variable KIF22 protein levels in KI67^+^ cells, we hypothesized that KIF22 protein levels change throughout the cell cycle. To test this, we quantified nuclear immunofluorescence intensity of KIF22 and KI67 in two 12 PCW brains (see methods for details of sampling procedure). KI67 protein levels vary during the cell cycle: lowest in G1 phase, increasing through S and G2 to peak in mitosis (Fig. 5*o*) (Scholzen and Gerdes 2000; Miller et al. 2018). We found a strong correlation between KIF22 and KI67 intensity (Brain 1 rostral *R*^2^ = 0.8095, middle *R*^2^ = 0.8139, and caudal *R*^2^ = 0.6691. Brain 2 rostral *R*^2^ = 0.7489, middle *R*^2^ = 0.7447, caudal *R*^2^ = 0.7763) (Fig. 5*d*–*f*). To ensure the correlation observed was not a result of nucleus size changing with cell cycle, we confirmed that KIF22 protein levels did not correlate with nuclear size by DAPI staining (Brain 1 rostral KIF22 *R*^2^ = 0.104, middle KIF22 *R*^2^ = 0.0874, caudal KIF22 *R*^2^ = 0.2969. Brain 2 rostral KIF22 *R*^2^ = 0.1183, middle KIF22 *R*^2^ = 0.0512, and caudal KIF22 *R*^2^ = 0.2287). A strong positive correlation was also observed at 14 PCW (Brain 1 rostral *R*^2^ = 0.7465, middle *R*^2^ = 0.6668, caudal *R*^2^ = 0.634, Fig. 5*j*–*l*). Again, we confirmed that KIF22 protein levels did not correlate with nuclear size (rostral KIF22 *R*^2^ = 0.1239, middle KIF22 *R*^2^ = 0.0599, caudal KIF22 *R*^2^ = 0.0229). This demonstrates that the correlation between KI67 and KIF22 is consistent between ages and rostral-caudal location. Combining all values of KI67/KIF22 nuclear intensity values showed that KIF22 was expressed at significantly higher levels in KI67^+^ cells (Fig. 5*m*) with a strong correlation (*R*^2^ = 0.7236) between KIF22 and KI67 levels (Fig. 5*n*). Although KIF22 expressing cells were scattered throughout the VE, VZ, and SVZ there was a general trend for the cells expressing the highest levels of KIF22 to be closest to the apical surface (yellow colored dots on scatterplots Fig. 5*d–f,j–l,n*) with lower expressing cells tending to be further from the apical surface (blue colored dots on scatterplots Fig. 5*d*–*g*, *j*–*l*,*n*). During interkinetic nuclear movement radial glial cell nuclei move to the apical surface to perform mitosis so this spatial distribution suggests KIF22 is expressed at high levels by radial glial cells undergoing mitosis at the apical surface of the VZ. Cerebral cortex progenitor that do not undergo interkinetic movement (intermediate progenitors and outer sub-ventricular zone progenitors) cannot be assigned to cell cycle phase using their position as we were able to do for radial glial cells which do undergo interkinetic movement. As *KIF22* transcript levels were at highest in all progenitors in M-phase (Fig. 1*h*) we suspect that cells expressing high levels of KIF22 and KI67 further away from the apical surface are cortical progenitors in M-phase not undergoing interkinetic movement but further experiments would be needed to show this.

From these data we show that, for radial glial cells, KIF22 protein levels change throughout the cell cycle in positive correlation with KI67: KIF22 is present in G1 and increases through S and G2 phase to peak in mitosis (Fig. 5*p*).

### ALDOA Protein is Highest in the Germinal Zones of the Cortex

**Figure 6:**
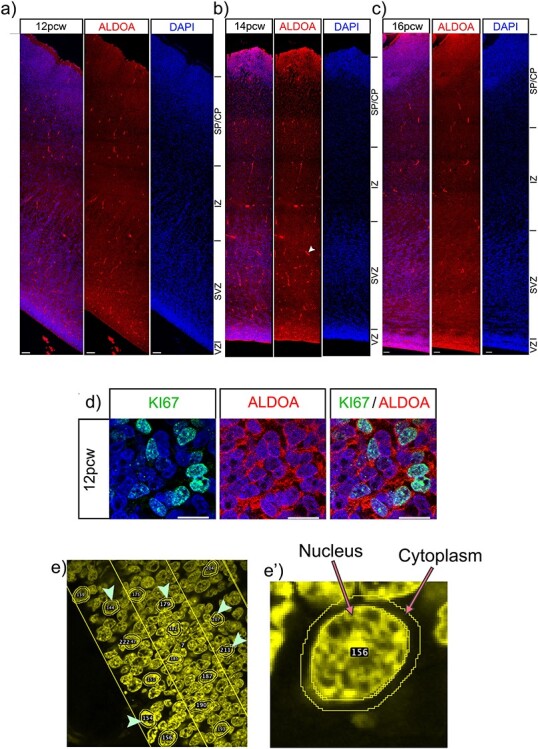
ALDOA protein expression in the cortex. ALDOA protein expression across the telencephalic wall at a) 12, *b*) 14 and *c*) 16 pcw. Scale bars = 100 μm. White arrow indicates non-specific binding to blood vessels. *d*) high magnification immunofluorescence of ALDOA and KI67 proteins, scale bar = 10 μm. *e*) low power image showing how cells were randomly selected for analysis using the DAPI channel. *e’*) high power image showing how the nucleus and cytoplasm were delineated for analysis

Bioinformatics analysis and in situ hybridisation show *ALDOA* mRNA levels decrease as progenitor cells move towards a neuronal fate (Fig. 1). Here we used immunofluorescence to characterize ALDOA protein expression across the telencephalic wall at 3 developmental time points; at 12, 14, and 16 PCW, ALDOA immunofluorescence is most intense in the VZ and SVZ before decreasing in the cortical plate (Fig. 6*a–c*). Double immunofluorescence for KI67 and ALDOA viewed at high magnification shows that ALDOA protein is primarily localized outside DAPI^+^ nuclei in the cytoplasm and that ALDOA is expressed by KI67^+^ progenitor cells and also by cells that do not express KI67 (Fig. 6*d*). The schematic (Fig. 6*e,e′*) illustrates the areas used for quantification of nuclear and whole cell ALDOA fluorescence presented in Fig. 6.

### ALDOA Protein Levels Do Not Correlate with Proliferation

**Figure 7:**
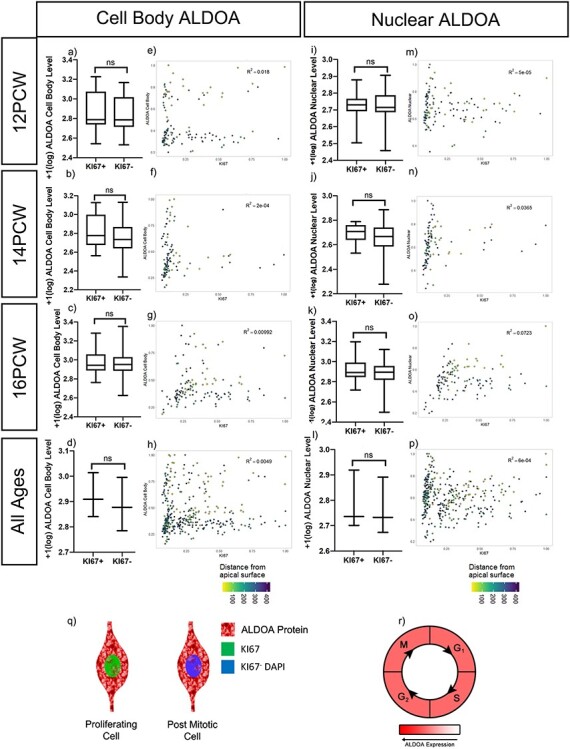
ALDOA protein quantification. (*a*–*d*) Cell body ALDOA protein fluorescent intensity in KI67+ and KI67− cells at (*a*) 12 PCW (raw data transformation = +1(log), bimodal distribution, Mann–Whitney test, p = 0.3702), (*b*) 14 PCW (raw data transformation = +1(log), normal distribution, unpaired *t*-test with Welch’s correction, *P* = 0.2032), (*c*) 16 PCW (raw data transformation = +1(log), normal distribution, unpaired *t*-test with Welch’s correction, *P* = 0.3523). (*d*) ALDOA cell body protein fluorescent intensity in KI67+ and KI67- cells, 12, 14, and 16 PCW individual datasets averaged, (raw data transformation = +1(log)), paired t-test, *P* = 0.0836. (*e*–*h*) ALDOA cellular protein intensity levels ls correlated to nuclear KI67 protein intensity at (*e*)12, (*f*)14 and (*g*)16 PCW with distance from ventricular edge indicated. (*h*) ALDOA whole cell protein intensity levels correlated to nuclear KI67 protein intensity pooled 12, 14, 16 PCW. (*i*–*l*) Nuclear ALDOA protein fluorescent intensity in KI67+ and KI67− cells at (*i*) 12 PCW (raw data transformation = +1(log), normal distribution, unpaired t-test with Welch’s correction, *P* = 0.7543), (*j*) 14 PCW (raw data transformation = +1(log), normal distribution, unpaired *t*-test with Welch’s correction, *P* = 0.0694), (*k*) 16 PCW (raw data transformation = +1(log), normal distribution, unpaired t-test with Welch’s correction, *P* = 0.0772). (*l*) ALDOA nuclear protein fluorescent intensity in KI67+ and KI67− cells, 12, 14, and 16 PCW individual datasets averaged, (raw data transformation = +1(log)), paired *t*-test, *P* = 0.1330. (*m*–*p*) ALDOA nuclear protein intensity levels is correlated to nuclear KI67 protein intensity at (*m*)12, (*n*)14, and *o*)16 PCW with distance from ventricular edge indicated. (*p*) ALDOA nuclear protein intensity levels correlated to nuclear KI67 protein intensity pooled 12, 14, and 16 PCW. (*q*) schematic demonstrating ALDOA protein is predominantly in the cytoplasm and lower in the nucleus in both KI67+ proliferating cells and KI67- post mitotic cells. (*r*) model showing ALDOA levels do not change with the cell cycle.

Although examination of ALDOA mRNA expression indicated it was enriched in progenitors we were unable to find a significant difference in ALDOA protein levels between KI67^+^ and KI67^−^ cells at 12 (Fig. 7*a*), 14 (Fig. 7*b*), and 16 PCW (Fig. 7*c*) in the human cortex. To look for any fluctuation in ALDOA levels with the cell cycle we quantified immunofluorescence for KI67 and cell body ALDOA (nucleus and adjacent cell body) using the same analysis as that described above for KIF22, and found no correlation or discernible pattern at 12 (Fig. 7*e*) (*R*^2^ = 0.018), 14 (Fig. 7*f*) (*R*^2^ = 2e-4) or 16 PCW (Fig. 7*g*) (*R*^2^ = 0.00992). These data show that in human cortex development, cellular ALDOA protein levels do not correlate with proliferation or fluctuate with cell cycle.

Previous work in different models demonstrated nuclear ALDOA level is greater in proliferating cells (Mamczur et al. 2010; Mamczur et al. 2013). To see if this was the case in human cortex development, we quantified nuclear ALDOA and KI67 (Fig. 6*e*) but found no significant difference in nuclear ALDOA fluorescence between KI67^+^ and KI67^−^ cells at 12 (Fig. 7*i*), 14 (Fig. 7*j*), or 16 PCW (Fig. 7*k*). We next tested if nuclear ALDOA levels in proliferating cells varied with cell cycle. Analysis of ALDOA and KI67 nuclear intensity established no correlation or pattern at 12 (Fig. 7*m*) (*R*^2^ = 5e-05), 14 (Fig. 7*n*) (*R*^2^ = 0.0365), or 16 PCW (Fig. 7*o*) (*R*^2^ = 0.0723). This shows nuclear ALDOA levels do not increase with proliferation, nor fluctuate with cell cycle. We combined results across the 12, 14, and 16 PCW. There was no significant difference between KI67^+^ and KI67^−^ cells when examining ALDOA protein intensity in the whole cell (Fig. 7*d*) or the nucleus (Fig. 7*i*). Using the pooled data, there was no correlation or discernible pattern when nuclear ALDOA intensity was graphed against nuclear KI67 level for the whole cell (Fig. 7h) (*R*^2^ = 0.0049) or the nucleus (Fig. 7*p*) (*R*^2^ = 6e-04).

## Discussion

### *16p11.2* Transcript Expression during Human Neurogenesis

The *16p11.2* CNV is a polygenic mutation that causes NDDs and the current study identified a number of the 29 *16p11.2* transcripts expressed in progenitor cells of the cerebral cortex. In addition to *ALDOA* and *KIF22* that are significantly enriched in progenitors, several other transcripts (e.g., *HIRIP3*, *PAGR1*, and *MAZ*) are also expressed in progenitors albeit at lower levels and are not significantly down-regulated as cells become postmitotic. The simultaneous expression of multiple *16p11.2* genes in cells undergoing neurogenesis suggests that these cells may be particularly vulnerable to simultaneous alteration in their dosage as a consequence of the *16p11.2* microdeletion or microduplication. This lends support to the hypothesis that neurogenesis is disrupted in *16p11.2* CNV patients and that this contributes to subsequent development of NDDs.

In this study we focused on the expression of *16p11.2* genes in progenitors of the developing cerebral cortex that will give rise to excitatory neurons. However, *KIF22*, *ALDOA*, *HIRIP3*, *PAGR1*, and *MAZ* transcripts are also expressed in the ventricular zone of the ganglionic eminences where the progenitors of the inhibitory interneurons that subsequently migrate into the cerebral cortex reside. Simultaneous increase or decrease of these transcripts in interneuron progenitors resulting from the *16p11.2* CNV may therefore have an impact on the neurogenesis or differentiation of interneurons.

### KIF22

KIF22 is a multifunctional protein that can regulate cell proliferation through at least two distinct mechanisms. First, KIF22 is a kinesin-like microtubule-based motor that binds microtubules and chromosomes during mitosis and regulates mitotic spindle microtubule stability and symmetric/asymmetric cell division (Tokai et al. 1996; Tokai-Nishizumi et al. 2005; Sun and Hevner 2014). Second, KIF22 regulates the expression of the cell-cycle regulator CDC25C. During cell division, CDC25C dephosphorylates CDK1, thus activating the CDK1-cyclinB complex while the CDK1-cyclin B complex phosphorylates CDC25C, causing an amplification loop to drive cells to mitosis (Nilsson and Hoffmann 2000). KIF22 directly transcriptionally represses CDC25C and inhibits mitosis; this transcriptional repression of CDC25C is dependent on KIF22 being phosphorylated on Thr463 (Ohsugi et al. 2003; Yu et al. 2014). KIF22 depletion in a tumor cell line accelerates the G2/M transition and slows M/G1 transition (Yu et al. 2014).

Overall, it therefore appears that KIF22 can act at several different points in the cell cycle making it difficult to predict how increased or decreased dosage of KIF22 in the *16p11.2* microduplication or microdeletion respectively would impact cell cycle in the specific context of cerebral cortex neural progenitors especially in light of the concomitant altered dosage of other *16p11.2* genes co-expressed with *KIF22* in progenitors. Our observation that *KIF22* mRNA and KIF22 protein levels both increase during the cell cycle to achieve the highest levels in G2/M phase that drop as cells enter G1 phase implies that KIF22 protein does not persist for long after it is translated and is degraded at the end of M-phase suggesting that both transcriptional and post-transcriptional mechanisms regulate its levels. A clear outcome of our study is that KIF22 levels positively correlate with KI67 in neural progenitors and steadily rise as the cell progresses through G1 > S > G2 > M phases culminating in the maximum level during M-phase. One possibility is that KIF22 is required to reach a threshold for mitosis to occur, after which its levels must decrease sufficiently to allow mitotic exit. Whether cells undertake proliferative or neurogenic divisions is a process heavily controlled by cell cycle length (Borrell and Calegari 2014). Perturbing *KIF22* gene dosage as a consequence of the *16p11.2* CNV might affect the timing of KIF22 protein reaching this threshold in neural progenitors and therefore affect cell-cycle kinetics and perturb neurogenesis and neuronal output. Our results suggest the hypothesis that KIF22 regulates neurogenesis in the human developing cortex through cell-cycle regulation.

### ALDOA

The process of brain development requires a vast and consistent supply of energy. Glucose is the predominant energy substrate for the fetal brain (Gustafsson 2009), therefore efficient and controlled glycolysis is essential for normal brain development. ALDOA is required for the fourth step of glycolysis, conversion of fructose 1,6-biphospate to dihyroxyacetone phosphate and gluteraldehyde 3-phosphate. The metabolic role of cytoplasmic ALDOA is well established, and ALDOA also has other non-glycolytic ‘moonlighting’ roles such as regulating mitochondrial function and cytoskeleton stability (Orosz et al. 1988; Pagliaro and Taylor 1992; Kao et al. 1999; Jewett and Sibley 2003; Buscaglia et al. 2006). In addition to its cytoplasmic roles, ALDOA has been observed in the nucleus (Mamczur and Dzugaj 2008; Mamczur et al. 2010; Mamczur et al. 2013) where it has been suggested to impact cell cycle by positively regulating cyclin D1 expression to mediate G1/S progression (Ritterson Lew and Tolan 2012; Fu et al. 2018). Cell-culture studies show ALDOA sub-cellular localization depends on the availability of energetic substrates, with addition of glucose driving ALDOA protein to the cytoplasm (Mamczur et al. 2013). Therefore, it is likely the primary role for ALDOA is metabolic when cells require, and have available to them, large amounts of energy. The majority of ALDOA studies have used highly abnormal cancer tissue, or artificial cell culture systems in which glycolytic enzymes have been shown to be increased (Ritterson Lew and Tolan 2012; Mamczur et al. 2013; Fu et al. 2018; Pollen et al. 2019). How these observations of ALDOA in a variety of systems relate to its role in human cerebral cortex development is unclear.

Altering ALDOA dosage in the developing brain will likely impact energy metabolism by altering the flow of metabolites through the glycolytic pathway and impacting subsequent pathways which feed on outputs of glycolysis. Disruption to energy metabolism during development has previously been linked to ASD and ADHD (Rash et al. 2018). The offspring of hyperglycemic mice presented microcephaly, a phenocopy of the microcephaly observed in *16p11.2* microduplication patients (Rash et al. 2018) and disruptions to energy metabolism may contribute to the microcephaly seen in the offspring of Zika infected mothers (Gilbert-Jaramillo et al. 2019). No homozygous null ALDOA patients have been identified suggesting it is essential for life, but patients with changes to ALDOA levels have been identified; one patient with reduced ALDOA activity presented microcephaly (Kreuder et al. 1996) and another presented intellectual disability (Beutler et al. 1973). Of particular interest is the finding of schizophrenia patients with upregulated cortical ALDOA levels (Beasley et al. 2006) and *16p11.2* microduplication is strongly associated with risk of schizophrenia. This information, coupled with our results that ALDOA is expressed in all cell types, make it clear that any changes to ALDOA dose will perturb energy metabolism at many stages in the brain, impacting its development.

ALDOA is much more abundant in the cytoplasm and we also found no clear relationship between cell body ALDOA levels and cell proliferation status. Nuclear ALDOA has been linked to cell proliferation (Mamczur et al. 2010; Mamczur et al. 2013; Fu et al. 2018) but we found no clear relationship between nuclear ALDOA protein levels and cell proliferation status. Although *ALDOA* mRNA levels are higher in proliferating cells compared to non-proliferating cells, quantitative analysis of ALDOA protein revealed that ALDOA protein persists once cells exit mitosis. Therefore, while ALDOA protein is abundant in progenitor cells of the developing human cerebral cortex and so may play a role in neurogenesis phenotypes, the persistent expression of ALDOA protein as cells become post-mitotic argues against a specific role in neurogenesis and raises the additional possibility that ALDOA also plays roles in differentiated neurons.

## Conclusion

Our study of *16p11.2* gene expression in developing human fetal cerebral cortex indicates that altered dosage of *KIF22*, *ALDOA*, *HIRIP3*, *PAGR1*, and *MAZ* caused by the *16p11.2* microduplication or microdeletion may impact on neurogenesis in the developing human cortex and we identified *KIF22* being a strong candidate for having a specific role in neurogenesis. Further studies are required to unpick the mechanisms involved, but given the nature of the tissue, the scope for studying this in vivo is currently limited. However, growth of new model systems in which *16p11.2* gene expression can be manipulated such as human cerebral organoids will provide the opportunity address these questions.

## Supplementary Material

CC_Morson_et_al_Supplementary_bhab067

## Notes

We thank Katherine Howe for her assistance with designing in situ probes and undergraduate student Emma Fowler (EF funded by a WR Henderson Scholarship) for her assistance with quantification.
