# Supplementary material for: Expression of Genes in the 16p11.2 Locus during Development of the Human Fetal Cerebral Cortex
Source: Cereb Cortex. 2021 Apr 7;31(9):4038–52. doi: 10.1093/cercor/bhab067 (PMC8328201; doi:10.1093/cercor/bhab067)
Supplement: CC_Morson_et_al_Supplementary_bhab067 [file cc_morson_et_al_supplementary_bhab067.docx]

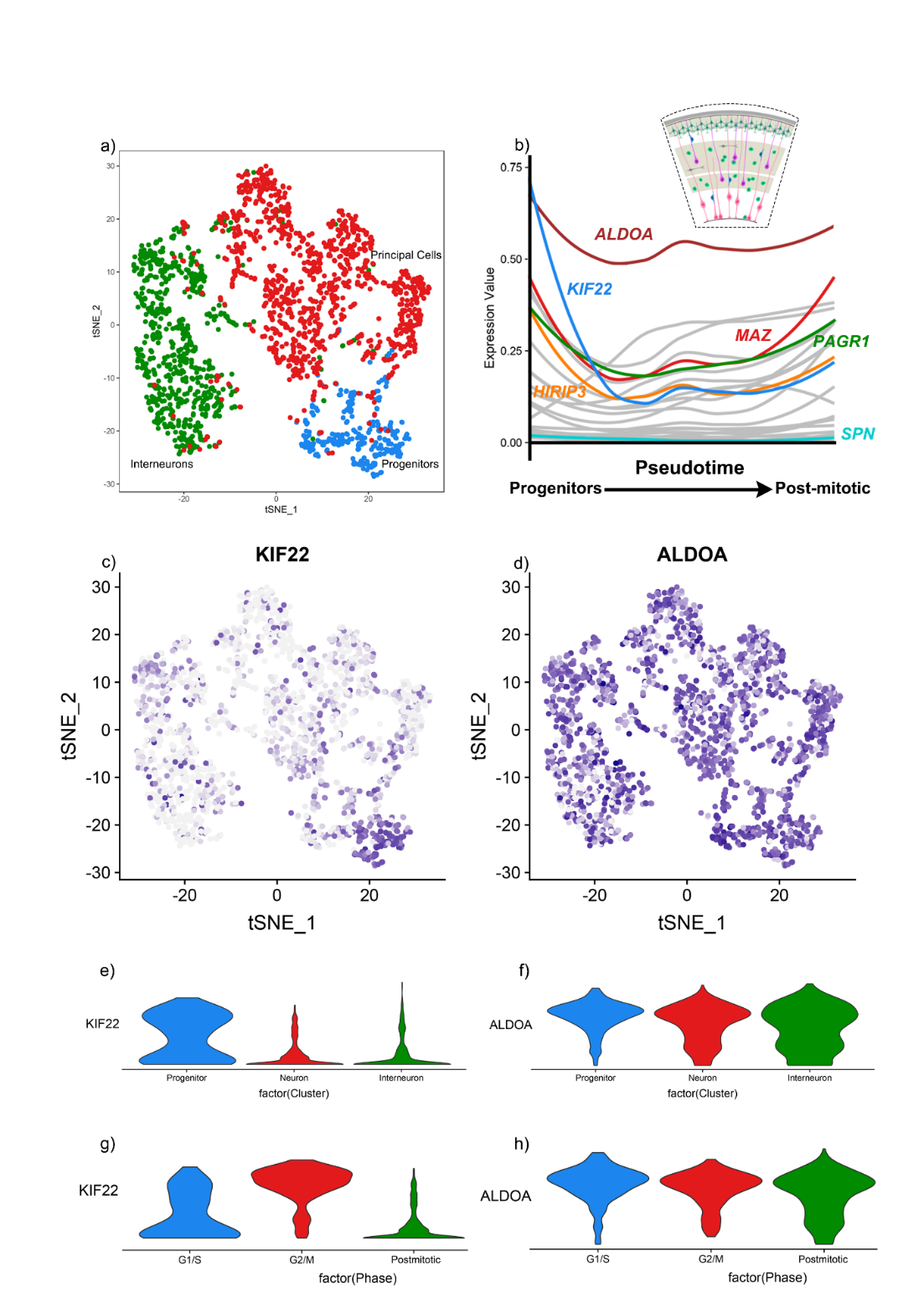


**Supplementary Figure 1: Bioinformatics analysis of Zhong et al scRNA-seq dataset which included all regions of the telencephalic wall in samples from 8-36GW.** a) tSNE clustering of cell types. b) changing mRNA expression levels of 16p11.2 genes as cells move from progenitors to neurons. Schematic of human cortex with dotted box indicates that all the regions of the cortex were used in this dataset. c) *KIF22* gradient plot. d) *ALDOA* gradient plot. e) Violin plots showing distribution of *KIF22* in different cell types. f) Violin plots showing distribution of *ALDOA* in different cell types. g) Violin plots showing distribution of *KIF22* at different cell cycle stages. h) Violin plots showing distribution of *ALDOA* at different cell cycle stages.
